# Alternative splicing in the genome of HPV and its regulation

**DOI:** 10.3389/fcimb.2024.1443868

**Published:** 2024-10-22

**Authors:** Yaping Wang, Fang Chen, Wenjie Qu, Yingxin Gong, Yan Wang, Limei Chen, Qi Zhou, Jiayin Mo, Hongwei Zhang, Lin Lin, Tianyi Bi, Xujie Wang, Jiashi Gu, Yanyun Li, Long Sui

**Affiliations:** ^1^ Department of Gynecology and Obstetrics, Obstetrics and Gynecology Hospital of Fudan University, Shanghai, China; ^2^ Shanghai Key Laboratory of Female Reproductive Endocrine Related Diseases, Shanghai, China; ^3^ Department of Obstetrics and Gynecology, Shanghai Changning Maternity and Infant Health Hospital, Shanghai, China; ^4^ Department of Obstetrics and Gynecology, Shanghai Pudong Hospital of Fudan University, Shanghai, China

**Keywords:** HPV, alternative splicing, splice sites, RBPs, splicing isoforms

## Abstract

Persistent infection with high-risk human papillomavirus (HR-HPV) is the main cause of cervical cancer. These chronic infections are characterized by high expression of the HPV E6 and E7 oncogenes and the absence of the L1 and L2 capsid proteins. The regulation of HPV gene expression plays a crucial role in both the viral life cycle and rare oncogenic events. Alternative splicing of HPV mRNA is a key mechanism in post-transcriptional regulation. Through alternative splicing, HPV mRNA is diversified into various splice isoforms with distinct coding potentials, encoding multiple proteins and influencing the expression of HPV genes. The spliced mRNAs derived from a donor splicing site within the E6 ORF and one of the different acceptor sites located in the early mRNA contain E6 truncated mRNAs, named E6*. E6* is one of the extensively studied splicing isoforms. However, the role of E6* proteins in cancer progression remains controversial. Here, we reviewed and compared the alternative splicing events occurring in the genomes of HR-HPV and LR-HPV. Recently, new HPV alternative splicing regulatory proteins have been continuously discovered, and we have updated the regulation of HPV alternative splicing. In addition, we summarized the functions of known splice isoforms from three aspects: anti-tumorigenic, tumorigenic, and other cancer-related functions, including not only E6*, but also E6^E7, E8^E2, and so on. Comprehending their contributions to cancer development enhances insights into the carcinogenic mechanisms of HPV and explores the potential utility of alternative splicing in the diagnosis and treatment of cervical cancer.

## 1 Introduction

Cervical cancer is the fourth most common cancer among women worldwide. In 2020, the disease resulted in over 300,000 deaths worldwide (Sung et al., 2021). HR-HPVs are the cause of the disease in most cases (Cohen et al., 2019). Hitherto, more than 400 HPV genotypes have been identified, and about 40 HPV types can infect the genital tract. Genital HPV can be divided into high-risk types (HR, which may cause invasive cancer) and low-risk types (LR, cause mainly genital warts) based on their oncogenic potential (McBride, 2022). Infection with HPV is usually transient and the majority of infections are cleared by the immune system (Stanley, 2012). In rare cases, infections with HR-HPVs may persist and lead to the development of cancer (Moscicki et al., 2012). Among approximately 15 HR-HPV types, which include HPV16, 18, 21, 33, 35, 39, 45, 51, 52, 56, 58, 59, 68, 73, and 82, HPV16 and HPV18 are responsible for more than 70% of global cervical cancers (Molina et al., 2024).

HPV is a non-enveloped DNA virus with a double-stranded genome containing around 8kb. The viral genome is separated by two polyadenylation (pA) signals, viral early (pAE) and viral late (pAL), into three parts: long control region (LCR), early region (E1, E2, E4, E5, E6, E7, E8) and late region (L1, L2) (Szymonowicz and Chen, 2020). The LCR contains most of the regulatory elements for viral DNA replication and transcription.

HPV infects undifferentiated basal cells through micro-wounds, activating the early promoter of episomal HPV DNA in the host nucleus to trigger transcription of early mRNA. The pre-mRNAs are polycistronic, undergo extensive alternative splicing, and are polyadenylated at the pAE site. This generates mature mRNA for early viral protein expression. As cells differentiate, the late promoter is activated and the viral life cycle enters a late stage. Similar to early gene expression, late mRNAs are generated through alternative splicing but polyadenylated at the pAL site (Burd, 2003; Doorbar et al., 2012). Therefore, the completion of the HPV life cycle and gene expression is inseparable from promoter switching, alternative splicing, and alternative polyadenylation sites. However, the small number of promoters somewhat limits the ability to fine-tune control of HPV gene expression at the transcriptional level (Bernard, 2013; Kajitani and Schwartz, 2020). In addition, the very compact genome and the absence of an internal ribosome entry site (IRES) in HPV RNA transcripts, which make all but the first ORF cannot be translated efficiently. So, HPV must evolve an efficient mode of RNA process, which can process polycistronic mRNAs for the expression of individual viral genes from the polycistronic RNA transcripts (Kozak, 1999). Besides, according to the translation scanning mechanism and 5’-cap dependent mechanism, the ribosome recognizes the strong Kozak start codon at the first ORF, initiating the translation of the first ORF. The ribosome falls off the mRNA after translation termination, so the strong Kozak start codon at the first ORF can efficiently block translation of downstream ORFs. Through splicing, the inhibition of the first start codon is removed and the downstream ORF is repositioned closer to the 5’ end of the mRNA, allowing downstream ORF expression (Kozak, 1992; Kozak, 2002; Zheng et al., 2004). The above two functions are mainly achieved by alternative splicing. So alternative splicing of HPV mRNAs following transcription is essential for the production of intact viral proteins. More importantly, relative levels of either early or late gene expression are regulated through alternative splicing, which affects the carcinogenic ability and infective ability of HPV. Therefore, even minor fluctuations in the efficacy of splicing could significantly affect the outcome of HPV infection (Johansson and Schwartz, 2013).

In recent years, the role of alternative splicing of tumor-associated viruses in the development of cancer has been increasingly emphasized, as it may offer potential targets for cancer therapy. Here, we will review and compare the alternative splicing events occurring in the genomes of HR-HPV and LR-HPV, with an update of the regulation of HPV RNA alternative splicing. In addition, many splice isoforms are produced during splicing, some of which with known coding functions. We will also summarize the function of these splice isoforms.

## 2 HPV alternative splicing in general

**Figure 1:**
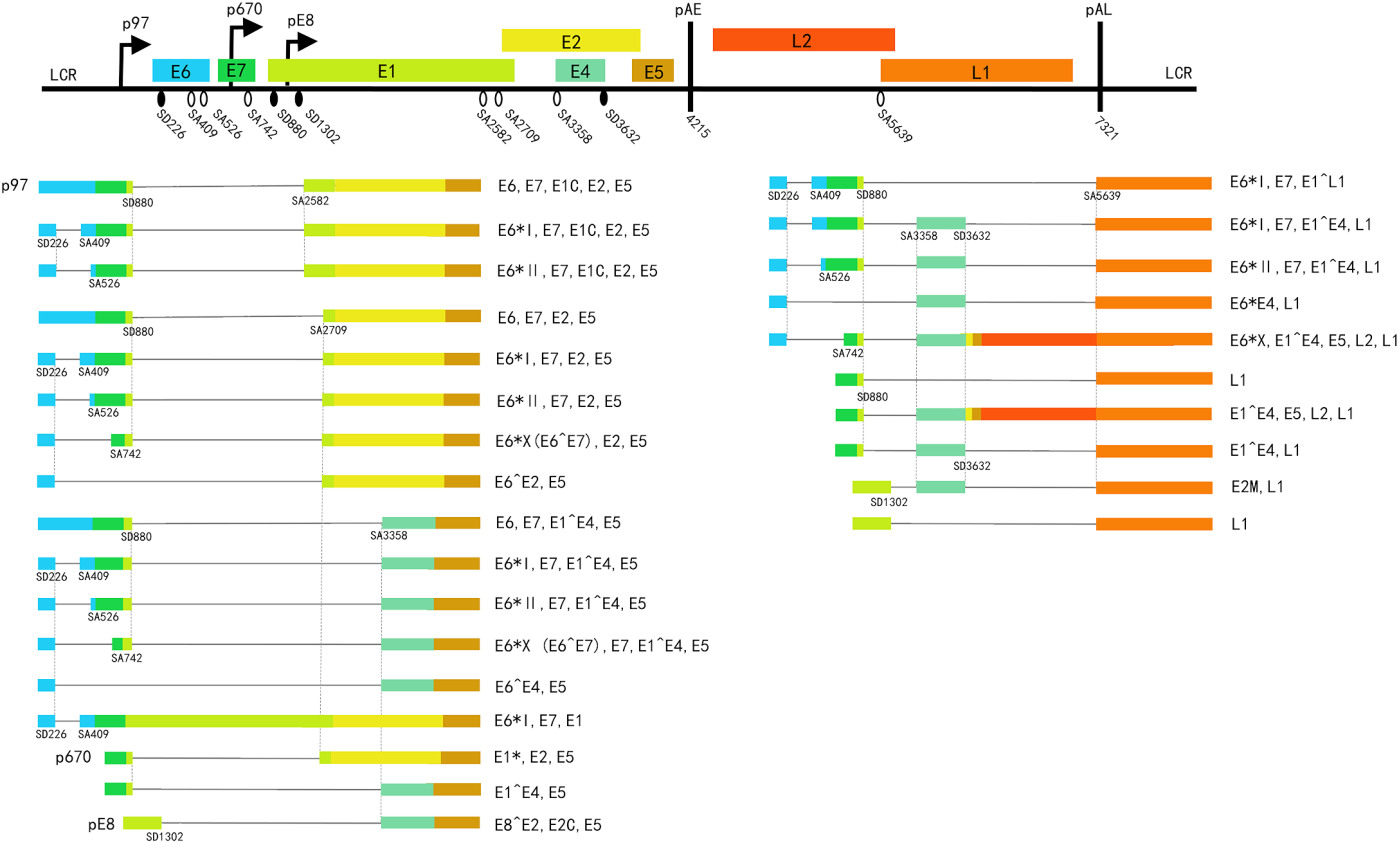
A schematic representation of the HPV16 genome and transcripts. The top part: linear genome, shows the eight open reading frames (ORF) (colored boxes), the three promoters (broken line arrow): p97, p670, pE8; and the early and late polyadenylation sites (thick black vertical lines): pAE and pAL; Black oval: 5’SS/splice-donor (SD). White oval: 3’SS/splice-acceptor (SA). Left lower part: early transcripts. Right lower part: late transcripts. Potential coding capacity is indicated to the right of each mRNA.

The process of removing introns from pre-mRNAs and connecting the remaining exons to produce mature mRNA is called splicing, while the different combinations of exons in the mRNA producing diversified mature mRNA are called alternative splicing (Bonnal et al., 2020). In HPVs, The mRNAs encoding E6 and E7 (Tang et al., 2006), E1 and E2 (Zheng et al., 2020a), as well as L1 and L2 mRNAs (Zhao et al., 2004; Dhanjal et al., 2015), are generated in a mutually exclusive manner from the same pre-mRNAs through alternative splicing. HPV mRNA splicing can be accomplished by an enzymatic machine termed the spliceosome inside the host cell nucleus (Will and Lührmann, 2011; Zhang et al., 2013; Graham and Faizo, 2017; Bowler and Oltean, 2019; Yang et al., 2019; Niño et al., 2022). The spliceosome is composed of 5 snRNPs (small nuclear ribonucleoproteins) (snRNPs: U1, U2, U4, U5, and U6 snRNPs) (Wahl et al., 2009; Hoskins et al., 2011). The spliceosome recognizes the junction between introns and exons by following the “GU-AG” rule and performs splicing (Faustino and Cooper, 2003; Hertel, 2008). Through alternative splicing, different mature mRNAs with different functions could be synthesized from a single gene, which increases the complexity of mRNA and the diversity of proteins (Zhang et al., 2021). The structure of spliced transcripts of different HPV types has been compiled by various laboratories (Van Doorslaer et al., 2013). HPVs’ transcription maps can be adopted from the PaVE (https://pave.niaid.nih.gov), these visions were updated in 2006 (Zheng and Baker, 2006). Later updates resulted in the current version of HPV16 (Chen et al., 2014; Yu et al., 2022) (**Figure 1**).

The process of alternative splicing is regulated by cis-acting elements and trans-acting factors. SR (serine and arginine-rich) proteins (Howard and Sanford, 2015) and hnRNPs (heterogeneous nuclear ribonucleoproteins) (Martinez-Contreras et al., 2007) are two essential auxiliary factors in enhancing or repressing splice site usage through the recognition of specific cis-acting RNA elements. In general, SR proteins play a positive role in splicing regulation and preferentially bind to exonic splicing enhancers (ESE) and intronic splicing enhancers (ISE). On the other hand, when hnRNPs bind to exonic splicing silencers (ESS) and intronic splicing silencers (ISS), it typically inhibits splicing (Busch and Hertel, 2012; Kędzierska and Piekiełko-Witkowska, 2017). Splicing efficiency determines the relative levels of viral proteins. Hence, tight regulation of splicing mechanisms must ensure adequate production of each HPV mRNA species and the optimal balance of viral proteins in HPV-infected and cancerous cells (Johansson and Schwartz, 2013).

## 3 Alternative splicing within the HR-HPV E6 and E7 gene region

### 3.1 Splicing events within the HR-HPV E6 and E7 gene region

The E6 protein is a high-risk factor for HPV-infected cells to become cancerous, while the E7 protein is the major driver of cell proliferation in infected cells (Roman and Munger, 2013). The combined action of both may lead to malignant transformation of the cells. In HR-HPVs, E6 and E7 genes are transcribed as a single polycistronic E6/E7 pre-mRNA from a single early promoter, which undergoes splicing to produce several transcripts. The E6E7 polycistronic pre-mRNA contains at least one donor and one acceptor splicing site that can trigger the splicing process, inducing the expression of a variety of E6 spliced transcripts termed E6* (Ajiro and Zheng, 2014). There are three 5′SS in the E6 ORF (SD226,SD221,SD174) and three 3′SS either in the E6 or E7 ORFs (SA409、SA526、SA742) in HPV16. In particular, nt 226 5’SS and nt 409 3’SS are preferentially selected for splicing. The preference for nt 226 is due to the relatively higher base-pairing affinity between U1 snRNP and nt 226 compared to other 5’SS (Ajiro et al., 2012). For the selection of 3’SS, the functional branch point sequence (BPS) at nt 385, along with the splicing enhancer upstream of nt 409 and its regulatory protein TRAP150, facilitate the preferential selection of nt 409 (Ajiro et al., 2012; Jönsson et al., 2024). BPS, located 15~40 nucleotides upstream of the 3′ss, is recognized by U2 snRNA during pre-mRNA splicing to facilitate splicing process (Brant et al., 2019a). Additionally, the proximal rule, which dictates the choice of nt 226 and nt 409 to excise the minimal intron length, is energetically most favorable as it requires the least amount of splicing energy (Reed and Maniatis, 1986). E6∗I and E6∗II, produced by splicing at SD226^SA409 and SD226^SA526, respectively, are the two main E6 isoforms expressed in cervical cancer (Chen et al., 2014; Cerasuolo et al., 2017). The expression level of E6∗II is regularly higher than that of the unspliced E6 mRNA but lower than E6*I. More evidence supports that the E7 oncoprotein is mostly produced from the translation of E6*I mRNA, perhaps because of the shorter upstream E6*I ORF being less hindering for translation initiation at the E7 ATG (Sedman et al., 1991; Tang et al., 2006; Brant et al., 2019a). In addition, splicing at SD226^SA742 and SD226^SA3358 are used to produce E6^E7 and E6*III, respectively. In other HR-HPV types, multiple splice sites and transcripts in E6 and E7 gene regions were identified. However, compared with HPV16 and HPV18, the research about other HR-HPV types is still limited. Previous reviews have summarized the known splice sites and E6/E7 mRNA variants of HR-HPV (Olmedo-Nieva et al., 2018; Zheng et al., 2022).

### 3.2 Functions of HPV E6/E7 splicing isoforms

Considering the differences in HPV gene expression at different stages of the lesion, scientists are dedicated to investigate the relationship between alternative splicing and lesion occurrence, and to find potential diagnosis and treatment targets. The specific HPV16 E6-associated transcription patterns and dominant transcripts changed as low-grade squamous intraepithelial lesions progressed toward cancer (Lin et al., 2015). The detection rate of the E6* I transcripts increases with the progression of SIL grades, and a further increase is observed in cervical cancer tissue (Chen et al., 2014; McFarlane et al., 2015; Brant et al., 2019b; Baba et al., 2020). Although the E6*I protein has not been detected in infected cells *in vivo*, it has been found that ectopic expression of E6*I has been shown to reduce tumor formation in cervical cancer xenografts in nude mouse models, indicating that the E6*I protein is biologically active *in vivo* (Filippova et al., 2014). Therefore, we believe that summarizing the functions of the E6*I protein is still necessary. The difficulty in detecting the E6*I protein *in vivo* may be due to its extremely short half-life, as documented in previous studies (Filippova et al., 2009; Paget-Bailly et al., 2019). Subsequently, more precise methods are needed to detect the E6*I protein in infected cells *in vivo* (Chen et al., 2014; McFarlane et al., 2015; Brant et al., 2019b). E6*I is a multi-functional protein that has been extensively investigated and can to some extent mimic E6 activity to accelerate the degradation of some PDZ-containing proteins(such as Akt, Dlg, and MAGI-1) in the absence of E6, but its role in cancer development is still controversial (Pim et al., 2009). Scholars have explored the function of E6*I from many aspects such as the p53 signaling pathway, apoptosis, cell polarity, oxidative stress, inflammatory response, and tumor resistance (Pim and Banks, 1999; Filippova et al., 2009; Pim et al., 2009; Williams et al., 2014; Artaza-Irigaray et al., 2019; Paget-Bailly et al., 2019). The results show that E6*I not only has an anti-tumor function but is also involved in the development of HPV-related cancer in some instances (Williams et al., 2014; Muñoz-Bello et al., 2018; Olmedo-Nieva et al., 2018; Artaza-Irigaray et al., 2019; Paget-Bailly et al., 2019).

E6*I can play an anti-tumor role by inhibiting p53 degradation, which may be achieved by being independent of E6 or by interfering with the oncogenic activity of the E6. HPV18 E6 indirectly promotes the expression of p14ARF through p53 degradation, while the overexpression of E6*I only induces a slight increase of the p14ARF (Vazquez-Vega et al., 2013). This result suggests that independent of E6, E6*I may affect p53 levels to prevent p53 from regulating p14ARF. On the other hand, E6*I protein can interact with full-length E6 and E3 ubiquitin ligase E6-associated protein (E6-AP) to prevent E6-mediated p53 degradation (Pim and Banks, 1999; D’Costa et al., 2012; Filippova et al., 2014). Except for the p53 pathway, the co-expression of E6 and E6*I promotes TNF-induced apoptosis (Filippova et al., 2009). While HPV16 E6 can accelerate the degradation of caspase-8, the E6*I could stabilize it by binding to caspase-8 at different sites than E6 (Filippova et al., 2007; Tungteakkhun et al., 2010; Manzo-Merino et al., 2014), which may provide a molecular explanation for the different effects between E6 and E6*I. Interestingly, HPV18 E6 and E6*I induce caspase-8 activation and its nuclear translocation, but not apoptosis. Possibly, nuclear translocation is beneficial for executing the viral life cycle or maintaining cell proliferation (Manzo-Merino et al., 2014). The effects of E6*I vary differently among different HPV types, resulting in a more intricate functional network for E6*I.

It is also important to note that E6* isoforms may cooperate with E6 in malignant progression in a manner not yet described. First, in the context of HPV-driven carcinogenesis, it has been proposed that E6*I-induced oxidative stress could cause genome instability and thereby facilitate the integration of HPV genomes into the host cell genome (Williams et al., 2011; Williams et al., 2014; Letafati et al., 2024). In line with this hypothesis, the correlation between the severity of cervical lesions and increasing levels of spliced E6*I mRNA was detected (Williams et al., 2014; Paget-Bailly et al., 2019). In addition, abnormal activation of the Wnt cell signaling pathway has been reported in HPV-related tumors (Bello et al., 2015). It was found that E6* and E6 cooperate to up-regulate TCF-4 transcriptional activity to promote the expression of Wnt target genes. Proliferation enhanced by β-catenin was increased when E6 and E6*I were co-transfected (Muñoz-Bello et al., 2018). These findings support that E6 and E6* synergistically activate the Wnt signaling pathway, thereby promoting malignant progression. A recent study demonstrated that the co-expression of E6 and E6* I promotes greater IL-6 overexpression (Artaza-Irigaray et al., 2019). E6*I may help promote a pro-inflammatory and highly proliferative microenvironment and contribute to cervical tumorigenesis. Interestingly, the relationship between E6*I and drug resistance has also been studied. The increased HPV16 E6*I can facilitate the drug-resistant phenotype, such as doxorubicin and etoposide (Wanichwatanadecha et al., 2012). These findings could provide a new perspective on the treatment of drug-resistant cervical cancer.

By investigating E6*’s function, it has been observed that E6* exhibits a seemingly paradoxical role, potentially linked to the E6/E6* pattern, yet the exact nature of its function remains an unresolved and intricate issue.

Studies on other E6 splice isoforms are limited. Regarding the differences in E6*II expression levels in the different lesion grades, conclusions were inconsistent among different studies. Some studies have found an increase of E6*II in high-grade lesions (Cricca et al., 2009; Pastuszak-Lewandoska et al., 2014), while a study has found a decrease of E6*II in high-grade lesions (McNicol et al., 1995). A study has proposed that the expression level of E6*II gene might be used as an indicator of cervical cancer severity (Pastuszak-Lewandoska et al., 2014) It should be noted that the study had a small sample size, and the results still need to be examined in larger patient cohorts. The E6*II protein was also shown to accelerate the degradation of p53 and had the opposite effect on cisplatin-induced apoptosis compared to E6*I (del Moral-Hernández et al., 2010; Vaisman et al., 2018). Consequently, it is necessary to investigate whether there is a relationship between its role in promoting apoptosis and the function of p53 degradation. Another isoform, HPV16 E6^E7, which can stabilize E6 and E7 oncoproteins via HSP90 and GRP78 (Ajiro and Zheng, 2015). E6^E7 is expressed at low levels, yet it may be a potent protein that can function well at a very low level. The functions of E6 isoforms are summarized in **Table 1**.

**Table 1:** Summary of the functions of splice isoforms.

Anti-tumorigenic functions			
Spliced Isoforms	Mechanism	Effect	References
E6*I	interfere with E6-mediated degradation of p53 by its binding to E6AP,E6 and to p53	growth arrest	(Pim and Banks, 1999; Pim et al., 2009; Martinez-Zapien et al., 2016)
	promote the overexpression of E-cadherin protein	cell adhesion	(Filippova et al., 2014)
	mildly increase the level of p14ARF independent of E6	growth arrest	(Vazquez-Vega et al., 2013)
	form a pseudo-DISC with E6 to promote TNF-dependent apoptosis	TNF-dependent apoptosis	(Filippova et al., 2009)
**E6^E7**	stabilize viral E6 and E7 oncoproteins via HSP90 and GRP78	E6 and E7 related functions	(Ajiro and Zheng, 2015)
Tumorigenic functions			
E6*I	decrease the levels of SOD2 and Gpx to increase ROS production	cell apoptosis HPV DNA integration	(Williams et al., 2014; Paget-Bailly et al., 2019)
	upregulate the Wnt/β-catenin cell signaling pathway through the TCF-4 transcriptional factor synergistically with E6	cell proliferation	(Muñoz-Bello et al., 2018)
	upregulate IL-6 expression independently of p53	pro-inflammatory and proliferative microenvironment	(Artaza-Irigaray et al., 2019)
**E8^E2**	bind to viral genomes and represses viral transcription and genome replication	Immune escape	(Kuehner and Stubenrauch, 2022)
Other functions			
E6*I	downregulate PDZ domain-containing proteins	loss of cell polarity and adhesion	(Pim et al., 2009)
	express alone	TNF-dependent apoptosis resistance	(Filippova et al., 2009)
	bind to the DED of caspase 8	caspase 8 stabilization, activation and its nuclear translocation	(Filippova et al., 2007; Tungteakkhun et al., 2010; Manzo-Merino et al., 2014)
	upregulate the level of AKR1C1 and AKR1C3	enhance chemoresistance	(Wanichwatanadecha et al., 2012)
E6*II	enhance the activity of caspase-9 and -3	cell apoptosis	(Vaisman et al., 2018)
	degrade p53 independently of E6	unknown	(del Moral-Hernández et al., 2010)
**E1^E4**	induce cell cycle arrest at G2/M	productive replication	(Wilson et al., 2007; Egawa et al., 2017)
	associate with keratin filaments and cause their reorganization	virus release	(McIntosh et al., 2010)
	inhibit SRPK1 phosphorylation	regulate alternative splicing and E2 function	(Prescott et al., 2014)

### 3.3 Regulation of alternative splicing within the E6 and E7 region

**Figure 2:**
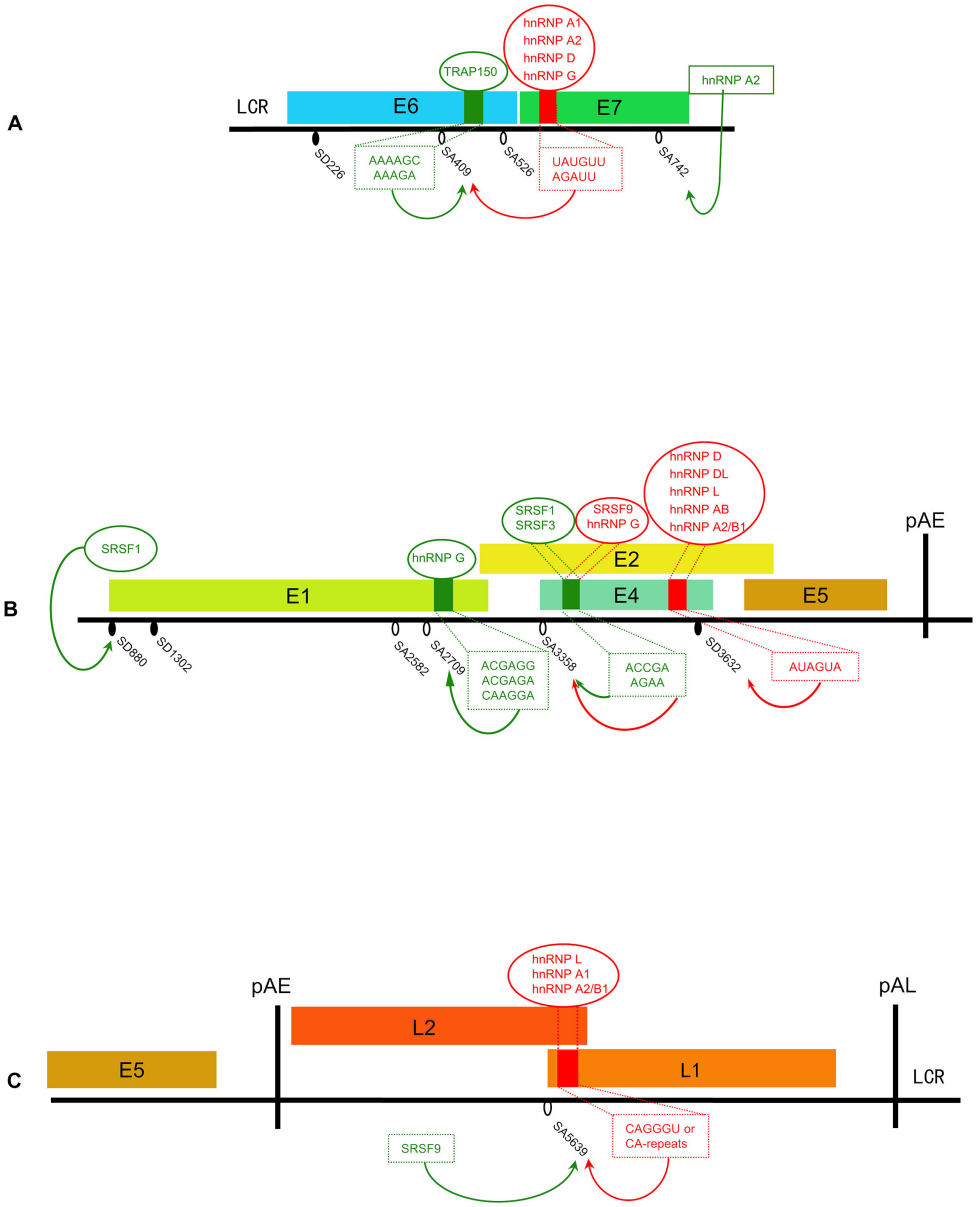
Schematic presentation of identified splicing factors involved in the splicing regulation of the HPV16 genome. **(A)** E6/E7 region. **(B)** E1/E2 region. **(C)** L1/L2 region. Small red squares on gene regions represent splicing silencers (SS) and small green squares represent splicing enhancers (SE); inhibitive splicing factors are shown in red, enhanced splicing factors are shown in green; and the sequence of the corresponding element below the cis-regulatory elements. Black oval: 5’SS/splice-donor (SD). White oval: 3’SS/splice-acceptor (SA).

The expression levels of E6, E7, and E6* proteins significantly influence the process of viral carcinogenesis, and the expression of these proteins is affected by splicing efficiency. Therefore, it is important to explore the regulatory mechanism of alternative splicing. E7 is derived from transcripts spliced within E6 ORF, and the production of E6 requires unspliced transcripts. Since E6 and E7 are both oncogenes, the regulation of the expression level of both genes must be stringent and maintained. The imbalance of E6 and E7 was found in infected cells showing senescence or apoptosis. A previous study suggested that splicing in the HPV16 E6 region is regulated by hnRNP A1 through the epidermal growth factor (EGF) pathway. It was shown that activation of the Erk1/2-kinase pathway promoted the retention of the E6 intron and the production of E6 mRNAs (Rosenberger et al., 2010). Further studies showed that hnRNP A1 and hnRNP D can bind to the splicing enhancer located in the E7-coding region of HPV16. This binding negatively regulates the splicing of 226^409, thereby promoting the production of E6 mRNAs and inhibiting the production of E7 mRNAs (Zheng et al., 2020b; Cui et al., 2022).The same conclusion was also obtained in HPV18 (233^416) (Ajiro et al., 2016). Even if hnRNP A1 is required to maintain the level of intron-retained E6 mRNA, the results from another study argues that there must be another role to control the action of hnRNP A1 and promote SA409 splicing to produce the appropriate amount of E7 (Jönsson et al., 2024). The research identified a novel splicing enhancer in the E6-coding region, located 35 nucleotides downstream of SA409. This enhancer interacts with TRAP150 to promote the splicing between SD226 and SA409, therefore ending up with more isoform of E6*I/E7 mRNA (Jönsson et al., 2024). In addition, hnRNP A2 interacts with the same splicing silencer as hnRNP A1 does to inhibit SA409 but is different from hnRNP A1 in that it redirects splicing to the downstream 3′SS (SA742) in HPV16 (Zheng et al., 2020b). Structural differences between the two proteins may explain their different effects on splicing (Zheng et al., 2020b). **Figure 2A** summarizes the identified splicing regulation within the E6 and E7 region. Current research has only identified some of the regulators, which are generally considered to have a singular function, that is, to either promote or inhibit splicing events. However, the true purpose of alternative splicing is to maintain a relative balance in the expression levels of E6 and E7 through these splicing events. To achieve this balance, there may be upstream regulatory mechanisms that modulate the activity intensity of factors that promote or inhibit splicing, but our understanding of these regulatory mechanisms is still quite limited at present. Exploring the upstream pathways of these regulatory factors is expected to resolve this problem.

## 4 Alternative splicing within the HR-HPV E1 and E2 gene region

### 4.1 Splicing events within the E1 and E2 gene region

E1 and E2 proteins play a crucial role in the initiation and regulation of HPV replication. In addition, the E2 protein is the negative transcription regulator of E6 and E7. Integration disrupts the E2 gene, resulting in increased expression of the E6 and E7 oncoproteins and cell transformation (Bhattacharjee et al., 2022). The generation of E2-coding mRNA involves splicing, specifically a splicing event that removes the E1-coding region (Kajitani and Schwartz, 2022). Similar to E6 and E7, the generation of E1 and E2 are mutually exclusive. In the HPV16 E1 and E2 gene region, there are three donor splicing sites (SD880, SD1302 and SD3632) and three acceptor splicing sites (SA2582, SA2709 and SA3358) (Van Doorslaer et al., 2013). SD880 is the most commonly used 5′ splice site (5′SS), which is used to generate E1^E4 mRNAs (880^3358) (Doorbar et al., 1990) and E2 mRNAs (880^2582, 880^2709) (Zheng et al., 2020a), respectively. After integration, a splicing event between SD880 with a nearby acceptor splicing site in the human genome leads to the generation of the fusion transcripts, which are used to produce E6*I and E7 (Brant et al., 2019b; Liu et al., 2023).

SA3358 in HPV16, recognized as the most commonly used 3′ splice site (3′SS), is efficiently utilized during both the early and late stages of the HPV16 life cycle. This site is used to generate HPV16 early mRNAs that encode the E6 and its splice variants E6*I, E6*II, and E6*III, as well as the E7 and E5, all of which are polyadenylated at the pAE. In the late stages of the viral life cycle, the majority of late pre-mRNAs are spliced from SD880 to SA3358 and polyadenylated at the pAE site to generate E1^E4 transcripts, a small fraction is polyadenylated at the pAL site to produce L2 transcripts or spliced from SD880 to SA3358 and then from SD3632 to SA5639, where they are polyadenylated at the pAL site to produce L1 transcripts (Doorbar et al., 1990; Somberg and Schwartz, 2010). E1^E4 has been demonstrated to induce G2/M cell cycle arrest, aid virus replication, and facilitate virus release (Wilson et al., 2007; McIntosh et al., 2010; Biryukov et al., 2017; Egawa et al., 2017). Thus, the E1^E4 protein functions as biomarkers indicative for active virus infection and the associated disease severity (Doorbar, 2013). In addition to these functions, E1^E4 has been shown to exert an inhibitory effect on the phosphorylation of SRPK1, a kinase involved in the regulation of SR protein functions (Prescott et al., 2014). Therefore, E1^E4 may be involved in the regulation of alternative splicing. SA3358 may be required for the production of E6, E6*, E7, E5, as well as E1^E4 and late proteins, while E1 and E2 expression is negatively affected by the efficient use of SA3358 (Li et al., 2013a). Other sites are used at relatively low frequencies. SD1302 is mainly used to produce E8^E2 mRNA (SD1302^SA3358), the precursor mRNA of which is generated from a separate promoter within the E1 gene (Stubenrauch et al., 2000; Lace et al., 2008). E8^E2 has been found to bind to viral genomes and represses viral transcription and genome replication (Dreer et al., 2016). Shortly after the virus infection, the level of E8^E2 determines whether the infection becomes latent or productive and during the productive phase, E8^E2 levels determine how much virus is produced (Dreer et al., 2016). Thus, E8^E2 is closely related to infection outcome. SD3362 is specifically used to produce late mRNA and will be discussed in the later section. Another two acceptor splicing sites, SA2582 and SA2709, are utilized at comparatively low frequencies. SA2709 is closer to the E2 ATG than SA2582, allowing SA2709 the preferred choice for producing transcripts encoding E2 compared to the suboptimal SA2582 (Zheng et al., 2020a).

### 4.2 Regulation of alternative splicing within the E1 and E2 region

As the most commonly used site, the regulation of SA3358 splicing has been extensively studied. There are many identified ESE sequences downstream of SA3358 that can directly interact with RNA-binding proteins. SRSF1 and SRSF3 are two splicing positive regulatory factors that have been extensively studied, And their transcriptional activity is regulated by the level of E2 protein (Johansson and Schwartz, 2013). In undifferentiated cells, low to medium levels of E2 protein result in enhanced transcription of SRSF1 and SRSF3. The high levels of SRSF1 and SRSF3 binding to corresponding ESE sequences promote the splicing of the SA3358 and concurrently inhibit the production of L1 mRNAs. However, this effect is significantly reduced when E2 level is high (Mole et al., 2009; Klymenko et al., 2016). Given that the E2 is typically peak in the late stage, it can be inferred that moderate levels of SRSF1 and SRSF3 in terminally differentiated cells lead to the production of L1 mRNAs (Rush et al., 2005; Jia et al., 2009; Somberg and Schwartz, 2010; Ajiro et al., 2016; Klymenko et al., 2016). E2 has the greatest transactivation effect on the expression of SRSF3, so differentially expressed SRSF3 controls the papillomavirus early-to-late switch (Graham, 2016). In contrast, SRSF9 and hnRNP G have been identified as inhibitors of splicing at SA3358. Specifically, SRSF9 inhibits splicing at SA3358 while redirecting splicing to SA5639 (Somberg et al., 2011). The binding of hnRNP G to the ESE downstream of SA3358 may inhibit exon inclusion between SA3358 and SD3632 (Yu et al., 2018). Most of the mRNAs encoding L1 protein contained the sequence between SA3358 and SD3632. Therefore, the role of hnRNP G may be to prevent the premature expression of late genes. In summary, SA3358 is effectively utilized throughout the early and late phases, with its regulation being influenced by varying concentrations of E2 and splicing regulatory factors, making the regulation at this site intricate.

Although SD880 is frequently utilized, there has been little research on its regulatory mechanism. It has been shown in RNA-mediated protein pull-down assays that interactions of splicing components(U1snRNP component U1-70K) with SD880 are under the control of the Akt kinase (Kajitani et al., 2017). Further investigation is warranted to elucidate the cis-acting elements and trans-regulatory factors at this site. Furthermore, the nucleotide around this site is recurrently present at the boundaries between HPV/human sequences after HPV integration (Brant et al., 2019b; Liu et al., 2023). It is an interesting question to ask if there are regulatory elements around the site and whether integration can affect the splicing of this site. Another site, SA2709, the splicing of which is regulated by hnRNP G and hnRNP D. hnRNP G binds to a splicing enhancer sequence in the E1 region, and this binding promotes the splicing of SA2709, leading to an increase in E2 mRNA production in HPV16 (Hao et al., 2022). In addition, hnRNP G is also involved in the regulation of splicing in the E6/E7-coding region (Hao et al., 2022). The timing of hnRNP G to perform these two different functions may depend on the state of cell differentiation. However, hnRNP D inhibits the splicing of 880^2709 and promotes the generation of E1 mRNAs (Cui et al., 2022). The contrasting regulatory roles of hnRNP D and hnRNP G in the production of E1 and E2 indicate the necessity of investigating the upstream regulatory pathway of both hnRNP D and hnRNP G proteins. This exploration is crucial to elucidate how the effects of them are properly controlled to produce appropriate levels of E1 and E2. The regulatory mechanism of SD1302 and SA2582 has not yet been explored. The regulation of these splicing factors is schematic in **Figure 2B**.

## 5 Late splice sites for the production of proteins L1 and L2

### 5.1 Splicing events between late splice sites

L1 and L2 are viral capsid proteins whose expression is suppressed at the early stage of infection and are expressed in highly differentiated epithelial cells during the late stage to assemble virions. The absence of L1 and L2 capsid proteins allows the virus to evade the immune system and persist, so suppression of L1 and L2 gene expression is a prerequisite for cancer progression and maintenance (Bodily and Meyers, 2005; Chow et al., 2010). The production of L1 and L2 proteins requires corresponding transcripts produced by alternative splicing of late pre-mRNAs. In addition, alternative splicing may be involved in the inhibition of these two protein expressions at the early stage of infection. SD3632 and SA5639 of HPV16 are dedicated to the generation of HPV16 L1 mRNAs. They are conditionally inactivated during the early stage of the HPV16 life cycle and activated during the late stage (Kajitani and Schwartz, 2020). It is worth noting that the intronic sequence between SD3632 and SA5639 encodes L2. The full activation of these two sites inhibits L2 mRNA production. As a result, the utilization of these two sites is strictly regulated to produce L1 and L2 at the late stage.

### 5.2 Regulation of alternative splicing at late splice sites

At the early stage of the life cycle, the utilization of SD3632 and SA5639 is suppressed (Johansson and Schwartz, 2013; Salma et al., 2016). For one thing, SD3632 lies between SA3358 and pAE, so SD3632 silence is crucial for early mRNA expression. In addition, regulatory elements upstream of SD3632 and downstream of SA5639 inhibit the activity of two sites. Also, many regulatory proteins that interact with these elements to regulate splicing at these sites have been identified. hnRNP D, hnRNP DL, hnRNP AB, and hnRNP A2/B1 are identified as suppressors of SD3632, which interact with splicing silencer elements located upstream of SD3632 (Li et al., 2013b; Li et al., 2013c; Dhanjal et al., 2015). hnRNP A1 interacts with a splicing silencer located downstream of SA5639 in the L1-coding region, leading to the inhibition of SA5639 splicing (Zhao et al., 2004). The phosphorylated hnRNP L binds to cis-elements around SD3362, SA5639, and pAE, resulting in the repression of splicing at both sites and promoting polyadenylation at pAE. Phosphorylation of hnRNP L is mediated by Akt kinase and the inhibition of Akt kinase will lead to dephosphorylate and induce the expression of viral late genes (Kajitani et al., 2017). Therefore, it can be speculated that Akt-related pathways play an important role in the regulation of late gene splicing.

At the late stage of the virus life cycle, repressive factors are relieved, and certain RNA-binding proteins bind to the early U-rich region (eUTR) upstream of the pAE, which inhibits the activity of pAE and activates these two late sites, SD3632 and SA5639, for the expression of late proteins. In addition to these regulatory factors, DNA damage response (DDR) is also involved in the regulation of late gene splicing (Nilsson et al., 2018b). hnRNP C was recruited to the HPV16 DNA by the DDR factors, and this recruitment increased the chances of hnRNP C binding to newly synthesized mRNAs and polyadenylation factors, thereby inhibiting pAE and activating SD3632 splicing (Nilsson et al., 2018a). Strongly related to the DDR factor BCLAF1, TRAP150 responds to DNA damage by recruiting U2AF65 and enhances the late mRNA splicing, which leads to late gene expression (Nilsson et al., 2018a). As mentioned earlier, TRAP150 also enhances the splicing of E6*I. Higher staining of TRAP150 was observed in the basal and middle cell keratinocyte layers than in the upper differentiated cells (www.proteinatlas.org). Similar to many SR proteins, TRAP150’s function may be related to its expression level.

Importantly, SD3632 and SA5639 cannot be activated completely considering the necessity of L2 mRNA production, since the intronic sequence between SD3632 and SA5639 encodes L2 (Kajitani and Schwartz, 2020; Kajitani and Schwartz, 2022). The regulatory mechanisms that control the activation of the two sites to produce L1 and L2 proteins simultaneously are not well understood. Additionally, the utilization of SD3358 has been found to exert an inhibitory effect on both SD3632 and SD5639 (Li et al., 2013a). However, during the late stage of the viral life cycle, the concurrent activation of all three sites is essential (**Figure 1**). These enigmatic regulatory factors constitute a complex regulatory network of late gene expression. The control of late gene expression is important for the establishment of the persistence of HPV infection. Therefore, it is meaningful to identify the factors that control the expression of late genes in HPV. The regulation of these splicing factors is schematic in **Figures 2B, C**.

## 6 Splicing within LR-HPV

Compared to HR-HPVs, research on alternative splicing of LR-HPVs is very limited. The main focus was on HPV11 and HPV6, and transcription from cell lines and benign condyloma acuminatum was mapped. It is widely known the E6 transcript from LR-HPVs does not have an E6 intron and thus does not undergo RNA splicing in the E6 coding region (Mesplède et al., 2012). The transcription of the E6 mRNAs and E7 mRNAs of LR-HPV were started from two different early promoters. Notably, the splicing of the E6 intron might serve as a key event for the expression of biologically active E1 protein (Remm et al., 1999; Hubert and Laimins, 2002). However, the E6 transcripts from LR-HPVs do not undergo splicing in the E6 coding region, effective expression of E1 protein may be achieved through the use of other regulatory factors within the E6 and E7 regions (Isok-Paas et al., 2015). In the E1 and E2 gene region, three splicing donor sites SD847, SD1272, SD1459 and three splicing acceptor sites SA2622, SA3325, SA3593 have been identified, corresponding to SD880, SD1302 and SA2582, SA2709, SA3358 in HPV16, respectively. Similar to HPV16, splicing at 847^3325 is used to produce E1^E4 transcripts and 1272^3325 to E8^E2 transcripts. HPV11 E8^E2 proteins also inhibit viral DNA replication (Isok-Paas et al., 2015). SD3593 and SA5771 are used for HPV11 late protein production. In addition, the transcription pattern of HPV6 identified in HPV6-positive condyloma acuminatum samples was similar to that of HPV16 in HPV16-positive CIN2 (Chen et al., 2014). Therefore, LR-HPVs and HR-HPVs appear to have similar splicing patterns except for splicing differences in the E6E7 region. The unique splicing in the E6E7 region of HR-HPVs and enriched E6* expression in HR-HPV-related cervical cancer indicate that splicing in this region may be one of the mechanisms of HPV carcinogenesis.

## 7 Perspectives for further research on HPV alternative splicing

In the regulation of HPV alternative splicing, in addition to the regulatory proteins in the host cells, the viral proteins are also involved in this process. However, limited knowledge is known for the regulation of the viral proteins expression, the further understanding of which will be crucial for constructing a clearer virus-host interaction network. Current research on HPV RNA splicing is primarily focused on exploring single promoting and inhibiting mechanisms. However, the relative levels of viral proteins are essential for HPV carcinogenesis and the completion of the viral life cycle. Despite the unclear mechanisms that maintain this balance, disrupting the balance could be a promising therapeutic strategy for treating cervical lesions. Furthermore, SR proteins and hnRNPs, involved in the regulation of alternative splicing, can be used as therapeutic targets. Several splicing factor inhibitors are being tested in pre-clinical and clinical trials in other cancers. In addition, the relationship between HPV alternative splicing and tumor immunity has been noted (Li et al., 2018; Jiang et al., 2022), but more in-depth studies are still needed. Exploring the relationship between HPV RNA alternative splicing and tumor immunity will be helpful to improve the implementation of immunotherapy. At last, current research mainly focuses on HPV16 and 18, and in some areas, some non-16/18 types are more prevalent, such as HPV31, 33, 35, 39, 45, 51, 52, 56, 58, and 59, and other probable HR-HPVs. It would be interesting to study these types of alternative splicing. Because different types have different oncogenic potentials, and the reasons for this are not entirely clear. Understanding the alternative splicing characteristics of these types can provide part of the explanation for this issue.
